# Determinants of influenza vaccine hesitancy among pregnant women in Europe: a systematic review

**DOI:** 10.1186/s40001-021-00584-w

**Published:** 2021-09-28

**Authors:** Gbadebo Collins Adeyanju, Elena Engel, Laura Koch, Tabea Ranzinger, Imtiaz Bin Mohammed Shahid, Micheal G. Head, Sarah Eitze, Cornelia Betsch

**Affiliations:** 1 Centre for Empirical Research in Economics and Behavioural Science (CEREB), University of Erfurt, Erfurt, Germany; 2 Media and Communication Science, University of Erfurt, Erfurt, Germany; 3 Willy Brandt School of Public Policy, University of Erfurt, Erfurt, Germany; 4 Clinical Informatics Research Unit, Faculty of Medicine, University of Southampton, Southampton, UK

**Keywords:** Influenza, Vaccination, Infectious diseases, Pregnant women, Europe, Vaccine hesitancy, Vaccine refusal, Vaccine delay, Review, Maternal

## Abstract

**Background:**

Pregnant women are at high risk for severe influenza. However, maternal influenza vaccination uptake in most World Health Organization (WHO) European Region countries remains low, despite the presence of widespread national recommendations. An influenza vaccination reduces influenza-associated morbidity and mortality in pregnancy, as well as providing newborns with protection in their first months. Potential determinants of vaccine hesitancy need to be identified to develop strategies that can increase vaccine acceptance and uptake among pregnant women. The primary objective of the systematic review is to identify the individual determinants of influenza vaccine hesitancy among pregnant women in Europe, and how to overcome the hesitancy.

**Methods:**

Databases were searched for peer-reviewed qualitative and quantitative studies published between 2009 and 2019 inclusive. Databases included PubMed via MEDLINE, Cochrane Central Register for Controlled Trials, PsycINFO, SAGE Journals, Taylor and Francis and Springer nature. These covered themes including psychology, medicine, and public health. Following the Preferred Reporting Items for Systematic Reviews and Meta-Analyses (PRISMA) approach, 11 studies were eligible and analyzed for significant determinants of influenza vaccine hesitancy among pregnant women in Europe.

**Results:**

The most commonly reported factors were psychological aspects, for example concerns about safety and risks to mother and child, or general low risk perception of becoming ill from influenza. Doubts about the effectiveness of the vaccine and a lack of knowledge about this topic were further factors. There was also influence of contextual factors, such as healthcare workers not providing adequate knowledge about the influenza vaccine or the pregnant lady stating their antivaccine sentiment.

**Conclusion:**

Health promotion that specifically increases knowledge among pregnant women about influenza and vaccination is important, supporting a valid risk judgment by the pregnant lady. The development of new information strategies for dialogue between healthcare providers and pregnant women should form part of this strategy.

## Background

Seasonal influenza poses a threat to public health and puts a strain on health care systems each year. According to the World Health Organization (WHO), up to 20% of the global population can be infected with influenza each season [1]. The high rate of infections can result in many deaths and hospitalizations, especially during severe outbreaks [2]. In 2017, at least 650,000 deaths worldwide were associated with influenza, with an estimated 72,000 deaths in Europe [1]. One of the particularly vulnerable groups is pregnant women, as they are susceptible to an influenza illness [3], and increased risk of preterm birth and fetal death [4]. These risks are due to physiological and immunological changes during pregnancy that make women more sensitive to viral pathogens [5]. Within the population, pregnant women are at greater risk of influenza-associated morbidity and mortality [3, 4, 6].

A study considering 20,000 pregnant women over 6 years in the United States, Australia, Israel, and Canada, showed that there was a 40% reduction in hospitalizations from influenza in vaccinated individuals [7]. The European Centre for Disease Control has highlighted how pregnant women are among high-risk groups for severe influenza and hold a protective role for their unborn children and early births. The burden of influenza in infants can be greatly reduced by increased vaccination among pregnant women [8], reducing the risk of transmission to children during their first months of life.

Besides the risks to the mother, influenza can also lead to complications during pregnancy, which affect the health of the unborn child [3, 6, 9]. To prevent severe outcomes, influenza vaccinations are commonly recommended for pregnant women in their second or third trimester [3, 10]. Current research suggests that influenza vaccination presents no health risks to pregnant women and does not increase the risk of pregnancy complications [11]. Despite the risks of influenza and the positive impact of vaccination, one-third of pregnant women refuse to get vaccinated despite receiving the recommendation to do so, and only approximately half of eligible pregnant women received the influenza vaccine in 2018 [12–14]. In Italy, 96% of pregnant women went unvaccinated against influenza during the 2016–2017 influenza season, with noted contributory factors including drug refusal and the belief that there would be adverse events from vaccination [15].

According to the first comprehensive assessment of seasonal influenza vaccine coverage in the World Health Organization (WHO) European Region (2008/09 and 2014/15), influenza vaccination coverage has been declining among high-risk groups [16]. This hinders responsive preparedness and capacity to protect the population against recurrent influenza epidemics and may have a negative impact against other emerging outbreaks and public health emergencies, such as COVID-19.

Therefore, the goal of this review is to lay the groundwork for an evidence-based framework by identifying factors that drive influenza vaccine acceptance and demand among pregnant women in Europe. The findings can inform country- and regional-level policy decisions and complement health promotion activities.

## Vaccine hesitancy

Vaccine hesitancy has been identified as one of the leading factors that contributes to low vaccination coverage [17]. It was identified by the World Health Organization as “one of the 10 greatest threats to global health in 2019” [18], and has been an important topic across COVID-19 vaccine development and roll-out [19, 20]. People may accept some vaccines but reject others, and their beliefs may change over time. Hence, vaccine hesitancy is not always evident as a total refusal of all vaccines but as decision-making that can range between general rejection and acceptance of all vaccines [17, 21]. The WHO Sage Working Group defines vaccine hesitancy as the “delay in acceptance or refusal of vaccination despite availability of vaccination services” [21]. Vaccine hesitancy is complex and context specific, varying across time, place and vaccines [22].

Determinants influencing vaccine hesitancy are multi-dimensional and vary across vaccines and target groups [23]. It is therefore important to understand that determinants of vaccine hesitancy cannot necessarily be generalized across different vaccines and contexts, as barriers to vaccine uptake can vary [24]. Seasonal influenza requires annual vaccination, but in some countries it is recommended only for certain population groups and may therefore be associated with specific attitudes and myths. Hence, these factors should be considered when investigating influenza vaccine hesitancy [25]. The Theory of Planned Behavior provides a framework for considering psychological insights around vaccine hesitancy, as used for example by Schmid et al. [25]. They incorporated different clusters of determinants that influence vaccination intention and behavior. Therefore, determinants can be categorized into physical, contextual, sociodemographic, and psychological determinants to identify possible barriers to vaccine uptake.

## Objectives

Among the large body of evidence and numerous systematic reviews on influenza vaccine hesitancy [25–27], only a small number focus on pregnant women [28–30]. To the best of our knowledge, there is no specific European perspective on the topic. Analyzing the phenomena from a European perspective might offer relevant and geographically specific insights into vaccine hesitancy, as evidence suggests that it is particularly common in countries with well-established health systems [31]. As the Sage Working Group suggests, factors leading to low vaccine acceptance can help to partially explain low vaccination rates in countries where immunization is easily accessible for the population. In countries where access to healthcare and vaccination services is more limited, vaccine acceptance is probably not the main driving force behind low vaccination coverage [21]. It is therefore reasonable to assume that determinants of vaccination hesitancy and acceptance vary across countries and settings. Thus, the primary objective of the systematic review is to identify the individual determinants of influenza vaccine hesitancy among pregnant women in Europe. Based on the framework by Schmid et al. [25], the secondary objectives include the identification of specific factors that predict vaccine hesitancy, such as psychological, sociodemographic, physical, and contextual factors.

## Methods

### Eligibility criteria

The study characteristics are generally based on the Population/Intervention/Comparator/Outcome (PICO) approach [32]. The population under review (P) was pregnant women in WHO European Region member countries. Instead of an intervention (I), we assessed the determinants or factors influencing the outcome. There was no comparator (C), and lastly, the determined outcome (O) was influenza-vaccine hesitancy. Characteristics used as criteria for study eligibility are listed in Table 1. The main criteria were studies that focused on seasonal influenza vaccination, pregnant women in European countries, publications between 2009 and 2019 inclusive, and empirical studies that were peer reviewed and in English language. All studies relating to pregnant women and seasonal influenza vaccination were central to the review inclusion guideline.

**Table 1:** Inclusion and exclusion criteria for literature search

Study inclusion criteria	Study exclusion criteria
Referring to seasonal influenza vaccination	No reference to seasonal influenza vaccination
Focusing on factors or determinants of	No inclusion of determinants or
influenza vaccine hesitancy	factors of influenza vaccine hesitancy
Focusing on pregnant women	No focus on pregnant women
Focusing on European countries	No focus on countries in WHO European region
Published in English	Published in languages other than English
Published between 01.01.2009 and 11.30.2019	Published before or after 01.01.2009 and 11.30.2019
Primary studies	Secondary studies (meta-analysis or systematic
Peer-reviewed journal articles	Gray literature
Humans	Focusing on vaccine uptake rather than hesitancy

### Search and selection process

Table 2 shows the search terms used in this review. From these, a broad search string was developed and then adapted to all databases (see Appendix 1). This systematic review used databases in different areas to capture the great variety of aspects that define influenza vaccine hesitancy. The final search included the following databases and publishers: PubMed via MEDLINE, Cochrane Central Register for Controlled Trials, PsycINFO, SAGE, Journals, Taylor and Francis, and Springer Link. The initial search was conducted from 01.20.2020 to 02.15.2020.

**Table 2:** Keywords for literature search

Vaccin*	**AND**	Influenza*	**AND**	Pregnan*	**AND**	Hesitan*	**AND**	Determin*
Immuniz*		Flu		Girls		Behavior		Factor
Immunis*		Seasonal Influenza*		Women		Behaviour		Predict*
Inoculat*		Flu Shot		Ladies		Refus*		Delay
		Pandemic Influenza*		Expecting Mothers		Decision Making		Non-Acceptance
						Decision-Making		
						Choice*		
						Choose		
						Anti-Vaccine*		
						Concern*		
						Perception*		
						Confidence		
						Trust		
						Doubt*		
						Skepticism		
						Unsure		

**Fig. 1:**
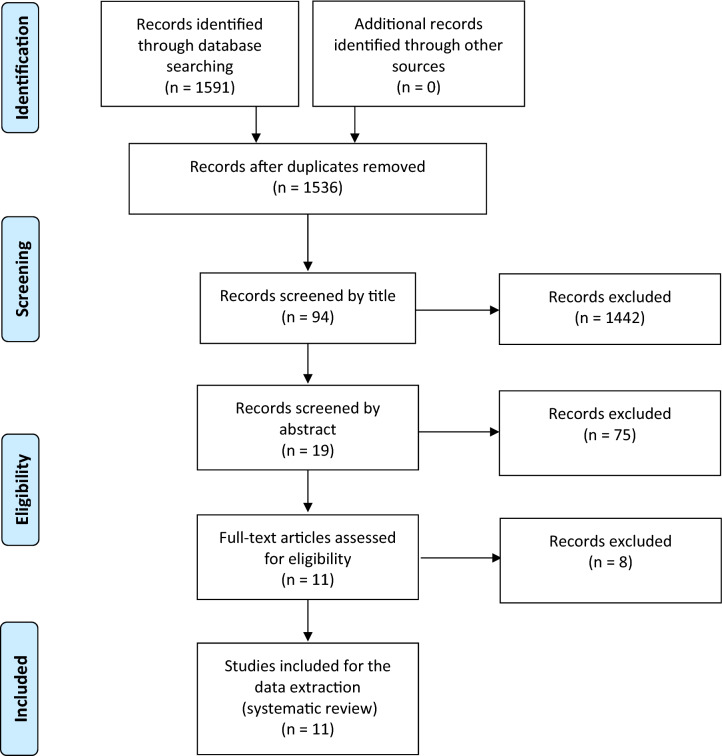
PRISMA flow diagram—study selection process

Guidelines for each database were created to ensure a systematic and transparent search procedure. These guidelines included a direct link to the website of the database, an adapted search string considering specific features and operators for each database, and the use of the inclusion and exclusion criteria, for instance, with filters. The analysis followed the PRISMA approach (Fig. 1). After duplicates were removed from the 1591 eligible papers, 1536 remaining articles were first scanned by title and abstract. Then, full texts of the 19 remaining articles were retrieved and assessed against the priori exclusion criteria, and 11 were finally included (Table 1; Fig. 1).

### Data extraction

Eleven studies met the inclusion criteria. For the data extraction, an Excel sheet was prepared by EE, LK, and TR, consisting of descriptive study characteristics, methodological aspects, and results of the studies (Table 3). The answer categories followed the theoretical framework discussed above. Determinants were considered important if the authors of the original papers listed them in their discussion of the results. The studies were distributed among EE, LK, TR, IS, and GCA to complete the Excel sheet.

**Table 3:** Data extraction

General information	Results
Authors of the study	Primary objective determinants
Year of publication	Psychological determinants
Country	Sociodemographic determinants
	Physical determinants
Method	Contextual determinants
Sample size and characteristics	Quality
Study design	Risk of bias/limitations
Focus: hesitancy or uptake	
	Reviewers’ assessment
	Authors’ recommendations
	Comments

### Synthesis of results

All data extracted were analyzed and used to inform classification of all determinants of influenza vaccine hesitancy, to address the goal of the review (see Table 4).

**Table 4:** Summary of findings

Authors	Year	Country	Sample size	Vaccine hesitancy measures used	Main determinants of influenza vaccine hesitancy
Blanchard-Rohner et al.	2012	Switzerland	261	Self-reported vaccine uptake (yes/no)	Lack of information by health care professionals Belief that vaccine is unsafe during pregnancy Anti-vaccine attitude
Bödeker et al.	2014	Germany	1030	Self-reported vaccine uptake (yes/no)	Lack of trust in vaccine Belief that vaccine is not necessary Lack of knowledge about the importance during pregnancy Anti-vaccine attitude
Bödeker et al.	2015	Germany	838	Self-reported vaccine uptake (yes/no)	That flu shot is not necessary Lacking awareness of influenza vaccination recommendations for pregnant women Mistrust in vaccine
Descamps et al.	2019	France	11,752	Self-reported vaccine uptake (yes/no)	Multiparity Less than postgraduate education
Maltezou et al.	2019	Greece	304	Self-reported vaccine uptake (yes/no)	Fear of adverse events (for them or the fetus) Influenza vaccination is not necessary No risk to get influenza Against all vaccinations
Maurici et al.	2015	Italy	309	Self-reported vaccine uptake (yes/no)	No need for the vaccination Opposition to vaccination Flu shot not recommended
O’Shea et al.	2018	Ireland	198	Self-reported vaccine uptake (yes/no)	Lack of recommendation by health care providers Lack of knowledge
Prospero et al.	2019	Italy	266	Self-reported vaccine uptake (yes/no)	Drug objection Low risk perception
Tuells et al.	2018	Spain	1569	Self-reported vaccine status	Unawareness of vaccine recommendation Belief that vaccination is not necessary
Vila-Candel et al.	2016	Spain	200	Medical records, immunization registry, and self-reported	Underestimation of personal risk Lack of information
Wilcox et al.	2019	England	314	Self-reported vaccine uptake (yes/no)	Concerns about side-effects Doubts about effectiveness Doubts about need

Overview of the studies considered in the systematic review. Authors, year of publication, country of study collection, and number of participants are listed. Measurements of vaccine hesitancy are listed. Determinants were considered important when the authors of the original papers listed them in their discussion of the results

### Quality assessment and risk of bias

Each study was assessed for risk of bias. The grading of the quality of each study was based on the study design using the Grading of Recommendations Assessment, Development, and Evaluation (GRADE) framework to either downgrade or upgrade the quality [33, 34].

### Interrater reliability

To assess the interrater reliability, from the 1,591 initially identified studies, 10 studies were chosen randomly. Each author of this systematic review rated the studies independently and decided whether the study should be included or excluded or whether there were any insecurities regarding this decision. The results of the rating are presented in Table 7 (see Appendix 3). Inconsistencies were discussed altogether to assure agreement and understanding of the selection criteria. The overall study interrater reliability Kappa coefficient was K = 0.93 (where 1.0 is a perfect score) and therefore justified the approach of the subsequent screening process, meaning each author received a defined number of studies to screen by title and abstract [35].

## Results

### Search result

Figure 1 presents the PRISMA flow diagram with the process of the selection of papers based on the four different phases: *Identification*, *Screening*, *Eligibility*, and *Including*. After removing the duplicates (55), 1536 potential studies remained. The remaining studies were screened based on title and abstracts. To decide if a study would be included or excluded, predefined eligibility criteria were applied. The screening resulted in the exclusion of 1442 studies, and therefore 94 studies remained. In the next screening comprising 75 studies, reasons for exclusion included: non-Europe region, not peer reviewed, pandemic H1N1 studies, study being a systematic review, etc. For the remaining 19 studies, full-text articles were retrieved and again assessed against the inclusion and exclusion criteria. As a result, 8 studies were excluded, and 11 studies met the final criteria. These studies were included in the data extraction. 

## Study *characteristics*

Within the timeframe of 2009–2019, 9 of 11 included publications were published between 2015 and 2019, one manuscript was published in 2012, and one in 2014. There were no studies from Eastern Europe. Publications mostly came from Northern, Western and Southern European countries, including Germany, Italy, Spain, Switzerland, Ireland, England, France, and Greece. All the studies were published in medical or public health-related journals, suggesting a potential lack of insight from other disciplines such as psychology and health communication. Ten of eleven studies used a cross-sectional non-experimental design, and in one case, a longitudinal study was conducted. Most of the studies followed a quantitative approach using standardized questionnaires. There were large differences in sample sizes between the studies, ranging from 198–11,752 participants (median participants 309, interquartile range 264–934). 

### Micro-level analysis of determinants

All identified determinants are classified into different factors, such as psychological, contextual, physical, sociodemographic, and others.

### Psychological factors

#### Influenza risk perception

Seven studies described how risk perception influences vaccine hesitancy [36–42]. A low-risk perception can result from the denial of the threats from an influenza infection, and the perception that the personal risk is low. In a study conducted by Vila-Candel et al. [41], 23% believed that they were not at risk of contracting influenza while being pregnant. Some women not only underestimated their personal risk of getting the flu, but also thought that the risks of adverse events from the vaccine were higher compared to the effects of an influenza infection [15]. In an Italian study, 48.3% of the women stated that getting vaccinated was not a priority for them [39].

#### Concerns about safety and risks of the vaccine

When it comes to influenza vaccination, many pregnant women had concerns about the safety of the vaccine. These concerns are related to the health of the mother as well as the unborn child, as pregnant women also fear that the vaccine could harm their children. These concerns are reflected in reported feelings of mistrust and insecurity. Five of the 11 studies identified these safety concerns as the most important factors contributing to vaccine hesitancy [36–38, 42, 43]. For example, a German study found that 60.4% of expectant mothers believe that the vaccine is unsafe during pregnancy and therefore mistrust the vaccine [36]. In a qualitative study conducted in Ireland, pregnant women who were hesitant to get vaccinated stated that they were afraid of possible risks of the vaccination, although unable to name specific dangers [44]. This shows that negative attitudes toward vaccination can result from poor knowledge or misconceptions.

#### Anti-vaccination attitude

Vaccine hesitancy can also result from negative attitudes toward vaccination in general. Maurici et al. [39] found that 28.8% of their sample refused all kinds of vaccinations for themselves or their children. While some women are opposed to vaccines in general, others specifically refuse to get vaccinated during pregnancy [43]. Furthermore, some women are susceptible to conspiracy theories that influence their decision against vaccination. In an Italian study, 6% of the women stated that vaccination is a business model motivated by the Pharma company’s desire for more profit [15].

#### Low vaccine effectiveness

There were also concerns associated with the effectiveness of the vaccination itself. Some women stated that the vaccine was not effective in preventing an influenza infection and therefore refused to get vaccinated [15, 42, 43].

### Contextual factors

#### Information and recommendations given by health care providers

We observed that barriers against vaccination during pregnancy on the contextual level are partly due to the lack of information and adequate recommendations by health care providers. As a result, a significant number of pregnant women do not know about the general influenza vaccine recommendation in pregnancy at all. Reasons for the lack of knowledge include that healthcare workers also lack knowledge around influenza vaccination, and that information about vaccine accessibility is not available [15, 43]. A Swiss study found a lack of support or recommendations from health care professionals that did not reflect the Swiss local public health guidance (43%) [43]. In a study conducted by Maurici et al. [39], 22.4% of the women sampled reported that the vaccine was not directly offered to them by physicians. Additionally, some health care providers even recommended against uptake of the influenza vaccine [39], although there was no clear medical or public health reason to do so.

The results show that not knowing about the recommendation to get vaccinated was a relevant factor. In a German study, 44.1% of the unvaccinated women were unaware of the official vaccination recommendation [37]. Tuells et al. [40] found that nearly 30% of pregnant women in Valencia, Spain, did not know about the recommendation to get vaccinated against influenza.

### Physical factors

#### Proximity of childbirth

One of the reasons for not getting vaccinated was proximity to childbirth. Even though influenza vaccination is recommended for women in their second or third trimester of pregnancy, some women fear that getting vaccinated then can be dangerous or that it is not necessary because childbirth is close [41]. An additional factor is multiparity, possibly resulting from experiences of not having been infected with influenza during prior pregnancies [45].

#### Prior influenza vaccination

Other reasons for refusal of vaccination included the individual’s experience with previous influenza vaccination and other vaccines. The experience of other people within the individual’s network who had bad experiences also impacts influenza vaccination during pregnancy [15, 36].

### Sociodemographic factors

Determinants for not getting vaccinated by sociodemographic status included being an ethnic minority, having a lower educational level, and being an immigrant or refugee [45].

### Other factors

Other factors mentioned were the need for time to think about a decision, as well as the fear of needles and drug objections [15].

### Quality assessment and risk of bias

To assess the quality of the included studies, an overall benchmark was made based on the study design; thereafter, the downgrade/upgrade framework was applied using GRADE indictors [34, 46]. Factors that described the quality of the study were assessed. Attention was paid to factors resulting in selection or reporting biases as well as sample size. The final quality assessment showed that six studies were of moderate quality and five were assumed to be of low quality (see Table 5). See Appendix 2 for definitions (High/Moderate/Very Low/Low).

**Table 5:** Quality assessment of studies

Author	Year	Study design	Sample size	Risks of bias	Overall quality
Blanchard-Rohner et al.	2012	Cross-sectional study, quantitative questionnaire	261	Selection bias: women without Swiss nationality were included in the sample	Moderate
Bödeker et al.	2014	Cross-sectional study, quantitative questionnaire	1030	Selection bias: overrepresentation of women who are generally more interested in health topics, exclusion of women with insufficient knowledge of German language Reporting bias: vaccination status was self-reported	Moderate
Bödeker et al.	2015	Longitudinal study, quantitative, standardized questionnaire	838	Selection bias: overrepresentation of women with a higher educational level, underrepresentation of women with immigration backgrounds Reporting bias: vaccination status was self-reported	Low
Descamps et al.	2019	Interview and medical records	11,752	Selection bias: underrepresentation of women with immigration backgrounds	Moderate
Maltezou et al.	2019	Cross-sectional study, quantitative, standardized questionnaire	304	Selection bias: low percentage of women in sample who were not vaccinated	Moderate
Maurici et al.	2015	Cross-sectional study, quantitative, standardized questionnaire	309	Selection bias: overrepresentation of women who were generally more interested in health topics Reporting bias: vaccination status was self-reported	Low
O’Shea et al.	2018	Cross-sectional study, qualitative, semi-structured interviews	198	Selection bias: exclusion of women with immigration background, underrepresentation of non-vaccinated women	Low
Prospero et al.	2019	Cross-sectional study, quantitative questionnaire	366	Selection bias: women who were generally more interested in health topics, high percentage of women in third trimester, overrepresentation of women with negative attitudes toward vaccination	Low
Tuells et al.	2018	Cross-sectional, descriptive study	1569	Selection bias not further specified	Moderate
Vila-Candel et al.	2016	Cross-sectional study, qualitative telephone interviews	200	Selection bias: overrepresentation of women with health-seeking behavior	Moderate
Wilcox et al.	2019	Cross-sectional study, quantitative and qualitative questionnaire	314	Selection bias: possibly missing subsets of population who were anti-vaccination Reporting bias: self-report of vaccine status	Low

## Discussion

This review summarized relevant determinants of influenza vaccine hesitancy that 11 qualitative and quantitative studies identified among pregnant women in Europe. The most frequently reported factors were psychological determinants, such as low risk perception, concerns about the risks and safety of the vaccine, poor knowledge, and anti-vaccine attitudes [36–40, 42]. Misperceptions about the vaccine exist in part because there is a lack of knowledge. Pregnant women are insufficiently informed about the risks of an influenza infection [36, 37, 40, 41, 44], which is directly related to existing negative attitudes toward vaccination [36–39, 43]. The lack of information or recommendations from health care providers is one of the factors that contributed to vaccine hesitancy [39, 43, 44].

To decrease vaccine hesitancy, it is essential to increase knowledge on several areas including—the seasonal influenza itself, vaccine safety and effectiveness, when flu shots are recommended, and risk perceptions of influenza among pregnant women in Europe. Therefore, the low risks associated with the vaccine (both for the mother and the unborn child) should be better communicated, along with increased awareness of the risks associated with an influenza infection. Primary caregivers play a crucial role in women’s decisions to get vaccinated because they are in direct contact with expectant mothers and can therefore provide the appropriate education. Health care providers should openly engage in discussions with pregnant women about vaccination and emphasize the evidence base around safety and effectiveness [42].

Vila-Candel et al. [41] stress that a combination of information materials and interpersonal recommendations from doctors or health care providers is the best solution to increase vaccine uptake. One of the problems identified here is the overall consistency and lack of information and recommendations from health care workers to pregnant women about the vaccination. Hence, interventions aimed at decreasing vaccine hesitancy should incorporate increasing knowledge and confidence of healthcare workers on the safety and effectiveness of vaccines and provide respective information material. To improve confidence, policy-makers should focus on interventions capable of invoking positive attitude toward vaccines, i.e., addressing issues such as vaccine adverse events, fear of needles, and disinformation about vaccines [24].

Mistrust and misinformation can be spread by healthcare providers. Thus, programs that assist health care providers in improving their vaccine communication skills as well as educate them about evidence-based responses to the most frequent concerns of pregnant mothers are necessary [47, 48]. The important role of healthcare providers in increasing vaccination demand needs to be adequately leveraged, because caregivers of infants are more likely to be nudged by physician recommendations or other trusted sources [49, 50].

Vaccine risk communication plays an essential role in addressing the psychological antecedence of vaccination behavior, especially risk perception [51]. Several studies have demonstrated risk beliefs and anticipated concern about vaccine-preventable diseases to correlate reliably with getting vaccinated [48]. Hence, proper identification of the causes of low-risk perception is important to design appropriate strategies. Those strategies are already applied in research on other target groups, such as childhood vaccination, HPV in adolescents, and vaccination in the elderly. Effective strategies that could be tested are, among others, education of health care workers for patient communication, evaluated misinformation debunking [52], and extended knowledge about preventable sequelae [53]. Also, although vaccine mandate has generated controversy overtime, but have been effective intervention toward population behavior change in some settings; hence, a consideration would be advised, especially for countries with high influenza infection rate with corresponding low vaccination demand [54].

Although influenza vaccination uptake in pregnant women differs across regions (US: 20%; Asia: 9.4–37.8%; South America: 3–97%; Australia 27%) [55–58], the reasons for vaccine uptake seem similar: confidence in their safety and effectiveness [25] and risk-perceptions for diseases and vaccines [59] are globally important. Most of the studies, worldwide and in this review, found several reasons for vaccine hesitancy, but only a few used systematically validated multidimensional models such as the 5C model [23]. Besides *Confidence* and *Complacency* (lack of risk perceptions), this model also includes *Constraints* (perceived structural barriers), *Calculation* (the process of information search during the vaccination decision), and *Collective Responsibility* (the value of community immunity and motivation to protect vulnerable others through one’s own vaccination). Explicitly, the last determinant could be vital for prospective mothers and should be explored in future research.

Hence, to achieve increased uptake and overcome the low demand for seasonal influenza vaccines among pregnant women in Europe, in line with this review’s primary objective, a stronger focus must be directed at addressing the identified individual determinants of influenza vaccine hesitancy.

## Limitations

This review has several limitations, including the quality of included studies. Majority of the studies assessed self-reported vaccination status. The quality of self-reported data can be limited due to social desirability and false statements; therefore, we cannot rule out that the results of the review were affected by reporting biases. The findings may have further been affected by selection bias. Within the studies, there was an underrepresentation of women with a migration status. Additionally, women with high educational levels and those who are generally more interested in health topics were overrepresented. Also, there was geographical bias: there were no studies from Eastern Europe, as most of the studies were conducted in Northern, Western or Southern European countries. Additionally, the restriction of the search strategy to only articles in the English language may have produced biased search results and affected the above because it excluded articles from journals in other languages. Therefore, all the above limitations indicate that more evidence is required around vaccine confidence in pregnant women from underrepresented regions and populations. We considered determinants where the authors of the included studies listed them in the discussion of their manuscripts, thus our findings may be influenced by the authors own perceptions of important determinants.

The systematic distribution of reasons for vaccination decisions identified here will allow interventions for pregnant women to be more targeted, and hopefully also more effective, in the future.

## Conclusion

This review highlighted several determinants of vaccine hesitancy in populations of pregnant women in Europe. Perceptions around safety issues and adverse events were common. We conclude that the education of healthcare providers is crucial to give stronger recommendations and address concerns effectively. Further research should focus on marginalized population groups, such as women with a migration background or other groups who struggle to access healthcare. Among these groups, specific social or cultural determinants may be particularly relevant. This review contributes to further research and practical applications of findings to address influenza vaccine hesitancy among pregnant women in Europe.

## Data Availability

The datasets generated and/or analyzed are available at OSF.

## Appendix 1: Search string

((Vaccin* OR Immuniz* OR Immunis* OR Inoculat*) AND (Influenza* OR Flu OR Flu shot OR Seasonal Influenza* OR Pandemic Influenza*) AND (Pregnan* OR Girls OR Women OR Ladies OR Expecting Mothers) AND (Hesitan* OR Behavior OR Behaviour OR Refus* OR Decision Making OR Decision-Making OR Choice* OR Choose OR Anti-Vaccin* OR Concern* OR Perception* OR Confidence OR Trust OR Doubt* OR Unsure OR Scepticism) AND (Determin* OR Factor OR Predict* OR Non-Acceptance OR Delay)).

## Appendix 2

See Table 6.

**Table 6:** Quality of evidence rating (Balshem et al. 2011)

Quality level	Interpretation
High	We are very confident that the true effect lies close to that of the estimate of the effect
Moderate	We are moderately confident in the effect estimate: the true effect is likely to be close to the estimate of the effect, but there is a possibility that it is substantially different
Low	Our confidence in the effect estimate is limited: the true effect may be substantially different from the estimate of the effect
Very low	We have very little confidence in the effect estimate: the true effect is likely to be substantially different from the estimate of effect

## Appendix 3

See Table 7

**Table 7:** Interrater reliability test

		Raters			
Study number	TR	LK	IS	EE	% agreement
#1	1	1	1	1	1.00
#2	1	1	1	1	1.00
#3	2	1	1	1	0.75
#4	1	1	1	1	1.00
#5	1	1	1	1	1.00
#6	1	1	1	1	1.00
#7	1	1	1	1	1.00
#8	0	0	0	0	1.00
#9	1	1	1	1	1.00
#10	1	2	2	1	0.50

Study interrater reliability K = 0.93

Interrater reliability: 0 = include, 1 = exclude, 2 = unsure

## Abbreviations

CEREB: Centre for Empirical Research in Economics and Behavioural Science
UNICEF: United Nations Children Emergency Fund
VPD: Vaccine Preventive Disease
WHO: World Health Organization
PRISMA: Preferred Reporting Items for Systematic Reviews and Meta-Analyses
PICO: Population Intervention Comparator Outcome
GRADE: Grading of Recommendations Assessment, Development, and Evaluation
HPV: Human Papillomavirus
CEREB: Center for Empirical Research in Economics and Behavioural Science
